# *Schistosoma japonicum* cathepsin L1: A potential target for anti-schistosomiasis strategies

**DOI:** 10.1371/journal.pntd.0013241

**Published:** 2025-07-07

**Authors:** Xianyu Piao, Yuanlong Wang, Ning Jiang, Pengfei Cai, Jiamei Duan, Shuai Liu, Qijun Chen, Nan Hou

**Affiliations:** 1 NHC Key Laboratory of Systems Biology of Pathogens, National Institute of Pathogen Biology, Chinese Academy of Medical Sciences & Peking Union Medical College, Beijing, China; 2 The Research Unit for Pathogenic Mechanisms of Zoonotic Parasites, Chinese Academy of Medical Sciences, Shenyang, China; 3 Molecular Parasitology Laboratory, QIMR Berghofer Medical Research Institute, Brisbane, Australia; 4 School of Biomedical Sciences, The University of Queensland, Brisbane, Australia; 5 State Key Laboratory of Respiratory Health and Multimorbidity, National Institute of Pathogen Biology, Chinese Academy of Medical Sciences & Peking Union Medical College, Beijing, China; 6 Key Laboratory of Livestock Infectious Diseases in Northeast China, Ministry of Education, Key Laboratory of Ruminant Infectious Disease Prevention and Control (East), Ministry of Agriculture and Rural Affairs, College of Animal Science and Veterinary Medicine, Shenyang Agricultural University, Shenyang, China; National Institutes of Allergy and Infectious Diseases, NIH, UNITED STATES OF AMERICA

## Abstract

**Background:**

To achieve sustainable and integrated control of schistosomiasis, it necessitates the implementation of comprehensive strategies, where effective vaccines could play a pivotal role. The limited identification and validation of schistosome antigens hinders the progress of vaccine development for the disease. Schistosome cysteine proteinases are considered as important targets for novel anti-schistosomiasis immunoprophylaxis due to their primary role in nutrient absorption. Previous research on the *Schistosoma japonicum* degradome has identified a group of cathepsin L-like proteases (SjCLs) that are up-regulated in hepatic schistosomula and adult worms.

**Methods/findings:**

In this study, five recombinant proteins representing the mature form of these SjCLs, designated as rSjCL1–5, were successfully produced. Mice immunized with the rSjCLs were subsequently challenged with cercariae to evaluate the immunoprotective efficacy of these proteins. The expression and localization of SjCL1 were analyzed by qRT-PCR, western blotting and immunofluorescence assays. Among these five rSjCLs, only the immunization with rSjCL1 conferred partial protection to the mice against *S. japonicum* infection, resulting in a reduction in worm burden by 34.9% ~ 38.0% and a decrease of egg burden by 46.2% ~ 48.3%. This immunization also effectively mitigated body weight loss and hepatomegaly in the challenged mice. SjCL1 was primarily localized along the intestinal intima of hepatic schistosomula, as well as male and female adults, and on the tegument of male adults. The mature form of SjCL1 was detected in the excretory/secretory products of the parasites. Hepatic schistosomulum treated with SjCL1 antibodies *in vitro* showed significant growth retardation, although remained viable and developed intestinal heme pigmentation, indicative of hemoglobin digestion.

**Conclusions/significance:**

Our study revealed that SjCL1 is essential for normal parasite growth and shed new light for the development of schistosomiasis vaccines targeting cathepsins, which play a key role in the early intra-mammalian stages of schistosomes.

## Author summary

Schistosomiasis is a neglected tropical disease caused by trematode worms of the genus *Schistosoma*. Anti-schistosomal vaccines for human or animal use is a critical part of a multifaceted control approach but is not available yet. Efforts are still needed to identify appropriate protective antigens to elicit effective immune responses. Cysteine proteases play crucial roles in nutrient absorption of trematodes and some of them have shown great value as a vaccine target. Cathepsin L-like proteases are important cysteine proteases discovered in *S. mansoni* and *S. japonicum.* While their enzyme activities have been reported, the role of SjCLs during infection has not been fully explored. In this study, we produced the mature form of SjCL1–5. Immunization with recombinant SjCL1 (rSjCL1) provided partial protection to mice against *S. japonicum* infection, whereas the other four rSjCLs did not confer any protective effect. SjCL1 was expressed in the intestinal intima of hepatic schistosomulum, male and female adults, as well as on the tegument of male adults. The mature form of SjCL1 can be detected in the products excreted or secreted by the larvae and adult worms *in vitro*. Overall, the findings indicate that SjCL1 holds potential as an immunoprotective target.

## Introduction

Human schistosomiasis is caused by three main species of the genus *Schistosoma*, including *Schistosoma mansoni*, *S. haematobium* and *S. japonicum*, with approximately 779 million people at risk of the disease [1,2]. The recently released World Health Organization road map for neglected tropical diseases sets out targets for schistosomiasis elimination as a public health problem by 2030 (78 countries validated for elimination as a public health problem, currently defined as <1% proportion of heavy intensity schistosomiasis infection) [3]. Of the three major *Schistosoma* species, the endemic region of *S. japonicum* is decreasing, primarily in Asian countries such as China and Indonesia [1,4]. Thus, the elimination of schistosomiasis japonica is most promising.

Extensive efforts have been made in schistosomiasis control mainly as the results of population-based preventive chemotherapy delivered through mass drug administration (MDA) of praziquantel (PZQ) [5]. However, PZQ is ineffective against juvenile schistosomes and does not prevent re-infection [6]. Consequently, the current strategies, which predominantly rely on the MDA programme, are insufficient for the complete elimination of schistosomiasis transmission [7]. Vaccines represent the most effective tools for long-term protection against infectious diseases. Integration of a clinically safe and effective vaccine along with MDA and other control measures may offer the best chance of achieving the goal of schistosomiasis transmission elimination. Currently, all schistosomiasis vaccines progressing to the clinical stages are against *S. haematobium* or *S. mansoni* [8–11], whereas the development of vaccines against *S. japonicum* is lagging behind.

Cysteine proteases contribute to pathogenesis in a variety of ways, including invasion, nutrition acquisition, immune evasion, and other host-parasite interactions [12,13]. Cysteine proteases play critical roles in extracellular proteolysis in parasites, whereas their mammalian counterparts are predominantly found in intracellular organelles [14]. These discoveries make proteases promising targets for the development of novel immunological or chemotherapeutic anti-parasite agents. Several types of cysteine proteases have been identified in *S. mansoni*, including cathepsin B (SmCB1, SmCB2) [15,16], SmCC [17], and SmCLs (SmCL1, SmCL2, SmCL3) [18–21], with various roles in the development of the flukes. In addition, SmCB1 has been shown to be essential for normal parasite growth in mammalian hosts, and vaccination with SmCB1 induced protection in mice challenged by *S. mansoni* [22–25]. The orthologs of *S. mansoni* cathepsins are also present in *S. japonicum*, including SjCB2 [26], SjCC [27], SjCL1, SjCL2 [28], and SjCL3 [29,30]. However, the protective function of these *S. japonicum* cathepsins against schistosome infection remains inadequately characterized.

Using whole-genome microarray analysis, our previous research on *S. japonicum* degradome revealed that a group of proteases exhibited increased expression in hepatic schistosomula and adult worms. Most of these proteases are cathepsins, including six schistosome cathepsin L-like proteases, which are likely to play important roles in the digestion of host blood proteins for both immature and mature parasites within hosts [31]. In this study, five recombinant proteins of these cathepsin L-like proteases were successfully generated and termed as rSjCL1–5. The immunoprotective effects of these proteins against *S. japonicum* infection were assessed. The study revealed the biological characteristics of SjCL1 and its potential as a vaccine target for schistosomiasis japonica.

## Materials and methods

### Ethical statement

All animal procedures in this study were conducted according to the animal husbandry guidelines of the Chinese Academy of Medical Sciences. The animal studies were reviewed and approved by the Experimental Animal Committee of the Chinese Academy of Medical Sciences, with Ethical Clearance Numbers IPB-2021–6.

### Parasites and animals

Parasites infected *Oncomelania hupensis* were provided by Jiangxi Provincial Institute of Parasitic Diseases, Jiangxi, China. Freshly released cercariae were harvested immediately after exposing the snails to light for 2h. For mouse challenge experiment, male and female *S. japonicum* infected snails were selected using duplex qRT-PCR, as previously described [32]. Single sex cercariae were released by single sex schistosome infected snails under light stimulation. Six-week-old male BALB/c mice and New Zealand white rabbits (pathogen-free, purchased from Vital River Laboratory Animal Technology Co. Ltd., Beijing, China) were percutaneously infected with cercariae (20 ± 1 male and 20 ± 1 female per mouse, 1000 ± 100 per rabbit). Sera from infected animals were collected on 42 days post-infection (dpi). Mice were infected with cercariae (100 ± 10 male and 100 ± 10 female per mouse) to generate hepatic schistosomula. Hepatic schistosomula (14 dpi), adult worms (42 dpi) and eggs (42 dpi) were manually isolated from the vascular system or liver tissues of infected mice as previously described [33].

### Phylogenetic analysis of cathepsin L sequences

Phylogenetic analysis of cathepsin L sequences was performed. Homologous CLs from *S. japonicum*, *S. mansoni*, *Homo sapiens* and *Mus musculus* were retrieved from NCBI database. Protein sequences were aligned using ClustalX 2.1. The alignments were further refined with GeneDoc software. The phylogenetic trees were constructed using MEGA11 with the neighbor-joining method. Bootstrap values were expressed as percentage of 1000 replicates. Putative glycosylation sites were predicted using NetNGlyc-1.0 [34].

### Recombinant protein production and polyclonal antibody generation

The His-tagged fusion proteins of mature SjCLs were prepared. The peptidase_C1 domain fragments of SjCL1 (GeneBank ID: AY814043.1, 788–1387 nt), SjCL2 (AY813594.1, 735–1348 nt), SjCL3 (FN314782.1, 370–1017 nt), SjCL4 (AY222874.2, 426–1034 nt) and SjCL5 (FN313884.1, 441–1070 nt) encoding genes were successfully amplified from schistosome cDNA using high fidelity Phusion DNA polymerase (Finnzymes Oy, Finland). Primers were designed using Primer BLAST, and listed in S1 Table. The clones were constructed using pEASAY-Blunt E1 Expression Kit (TransGen Biotech, Beijing, China). *E. coli* Transetta (DE3) (TransGen Biotech) was used to generate recombinant proteins with positive clones. Protein expression was induced with 0.5 mM IPTG at 16°C for 20 h. His-tagged fusion proteins were purified from inclusion bodies using Ni-NTA Agarose (QIAGEN) under denaturing conditions (8M urea in all buffers), following the manufacturer’s instructions. The protein solution underwent sequential dialysis against phosphate-buffered saline (PBS) buffer containing 6M, 4M and 3M urea (two changes). Proteins were analyzed by western blotting with monoclonal antibodies to the His-tag (Cell Signalling Technology, MA, USA). The concentration of these recombinant proteins was quantified by the BCA kit (Pierce, Rockford, IL, USA) in accordance with the manufacturer’s instructions, and and all the standard samples were prepared with buffer containing 3M urea. The final concentration of recombinant protein in the storage solution was adjusted to 1 mg/mL in 3M urea PBS buffer. The molecular weight of the proteins was predicted by the ProtParam tool. Rabbit polyclonal antibodies were prepared at Beijing Protein Innovation (Beijing, China) by immunizing New Zealand white rabbits. The immunogen was diluted to 500 µL with physiological saline, followed by the addition of an equal volume of Freund’s adjuvant. The rabbits were immunized with 400 µg recombinant proteins emulsified with complete Freund’s adjuvant for the first immunization, and 200 µg protein emulsified with incomplete Freund’s adjuvant every two weeks for three immunizations. The emulsion was administered subcutaneously along the back at 8–10 sites.

### Immunization and challenge experiments

The immunoprotective effects of SjCLs against schistosome infection were evaluated. BALB/c mice in SjCL1, SjCL2, SjCL3, SjCL4, SjCL5-immunized groups and control group were immunized with 60 µg recombinant proteins respectively or an equal volume of PBS (with 3M urea) emulsified with complete Freund’s adjuvant for the first immunization, and 30 µg protein or PBS (with 3M urea) emulsified with incomplete Freund’s adjuvant every two weeks for three immunizations. The immunogen was diluted in 170 µL physiological saline, and then emulsified with an equal volume of Freund’s adjuvant. The mixture was homogenized to form a stable water-in-oil emulsion, which was injected subcutaneously at 8–10 sites on the back and abdomen of the mice. Two weeks after last immunization, mice were challenged with cercariae (20 ± 1 male and 20 ± 1 female per mouse) released from infected *Oncomelania hupensis* snails as described in the Parasites and animals section. Adult worms and eggs were isolated and counted at 42 dpi. Two independent experiments were carried out, with 10 mice per group in each trial.

### Hepatic schistosomula, adult worms and egg recovery

Hepatic schistosomula and adult worms were manually isolated from the vascular system of infected mice using a stereomicroscope (Olympus Corporation, Tokyo, Japan). The schistosomal eggs were isolated by an improved sieving and enzymatic method [33]. In brief, livers derived from schistosome infected mice were chopped and homogenized in 500 ml ice-cold PBS. The suspension was successively passed through 80, 120, 160, 200, and 260 mesh metal sieves, and finally a 320 mesh nylon screen. The mixture was collected and washed three times by discarding the tissue debris-containing suspension after natural sedimentation on ice. The pellet was resuspended in 50 ml PBS containing 10 mg collagenase B, 125 mg trypsin, 10 µg penicillin, and 20 µg streptomycin, then incubated at 37°C for 3 h with gentle shaking. The sample was then centrifuged at 1,500 rpm at 4°C for 5 min, and the supernatant was removed. The egg pellet was then resuspended in 2 ml PBS and examined using a stereomicroscope.

### Total RNA isolation and quality control

Parasites of all stages were soaked in RNAlater solution (Ambion, CA, USA) overnight and stored at -80°C. Total RNAs were extracted using the RNeasy Mini kit (QIAGEN GmbH, Hilden, Germany) and the contaminating genomic DNA was completely removed from the RNA samples with TURBO DNA-free kit (Ambion). RNA quantification and quality control were conducted using a Nanodrop ND-1000 spectrophotometer (Thermo Fisher Scientific, Wilmington, USA) and denaturing agarose gel electrophoresis.

### Validation of SjCL1 transcription with Quantitative real-time PCR

QRT-PCR was carried out as previously described [35]. The primers for SjCL1, forward: 5’-TTTCCGTATTGTTGGCCCCC-3’, reverse: 5’-AAAGATGCCCTCCCGATAGC-3’, were designed using Primer BLAST (https://www.ncbi.nlm.nih.gov/tools/primer-blast/). The qPCR was performed using a Brilliant II SYBR Green QPCR master mix kit (Agilent Technologies, Carlsbad, USA), following the manufacturer’s instructions, on the 7500 Real-time PCR System (Applied Biosystems, Carlsbad, USA). The gene encoding the 26S proteasome non-ATPase regulatory subunit 4 (*PSMD4*, GenBank ID: FN320595) was employed as a reference control [36]. Reactions detecting the expression of glyceraldehyde-3-phosphate dehydrogenase (*GAPDH*, GenBank ID: FN324551) in standard cDNA (equally mixed cDNA from parasites at each developmental stage) were used as standard controls.

### Protein extracts and ES preparation

Parasites were homogenized using a mortar and pestle under liquid nitrogen condition and subsequently incubated with lysis buffer (8 M urea, 4% CHAPs, 1% DTT, 1% EDTA, 10 mM Tris, 35 μg/ml PMSF) for 30 min on ice, followed by centrifugation at 12,000 rpm for 30 min at 4°C. The culture medium was collected and stored at -80°C. Freshly isolated hepatic schistosomula (14 dpi, n = 100 per well), male adults (n = 20 per well) and female adults (n = 20 per well) were cultured in 24-well plates in RPMI 1640 for 8 h at 37°C and 5% CO_2_. The culture media were collected and concentrated using Amicon Ultra-15 Centrifugal Filters (Ultracel-10K, Merck Millipore, Carrigtwohill, IRL) to prepare schistosome excretory/secretory (ES) products that were then used for the detection of SjCL1. Protein concentration was quantified using a BCA kit (Pierce, Rockford, IL, USA) according to the manufacturer’s instructions.

### Western blotting

Recombinant proteins were analyzed by western blotting with monoclonal mouse antibodies to the His-tag (Cell Signalling Technology). The antigenicity of recombinant SjCL1 (rSjCL1), rSjCL2, rSjCL3, rSjCL4, and rSjCL5 was detected with sera from *S. japonicum* infected mice and rabbits at 42 dpi. Protein extracts and ES products from parasites were used to detect the presence of SjCL1. The proteins were separated by 12% SDS-PAGE gels and transferred to PVDF (polyvinylidene difluoride) membranes (Millipore, Bedford, MA, USA). The blots were blocked in a blocking buffer containing 5% skimmed milk for 1 h at 25°C. The monoclonal mouse antibodies to the His-tag (1:1,000 dilution), mice sera, rabbit sera and rabbit polyclonal antibodies against rSjCL1 were used as first antibodies (1:1,000 dilution) with normal IgG as control. Detection was made by incubation with first antibodies at 4 °C overnight, followed by incubation with IRDye 800 CW goat anti-mouse IgG (H + L) antibodies and goat anti-rabbit IgG (H + L) antibodies (1:10,000 dilution, all from Li-COR Biosciences) for 1 h at 25°C, respectively, using the Odyssey system (Li-COR Biosciences).

### Immunofluorescence microscopy

Localization of SjCL1 was determined by immunofluorescence. Serial cryosections (5–7 μm) were obtained and fixed for 10 min in 4% formaldehyde. Total IgG from sera of rabbits immunized with rSjCL1 was purified using the rProtein A Sepharose Fast Flow Kit (GE Healthcare, Uppsala, Sweden) according to the manufacturer’s instructions. After blocking with 5% BSA buffer (in PBS) for 2 h at 25°C, the sections were incubated with rabbit polyclonal antibodies against rSjCL1 (2 mg/mL, 1:500 dilutions) and IgG from a non-immunized rabbit in blocking solution overnight at 4°C, followed by incubation with Alexa Fluor 555 donkey anti-rabbit IgG (H + L) (1:1,000 dilution, Invitrogen) for 1 h at 25°C. The sections were finally stained with 50 μL Fluoroshield Mounting Medium with DAPI (Abcam) and visualized immediately with a TCS SP5 confocal microscope (Leica Microsystems, Wetzlar, Germany).

### *In vitro* cultivation of parasites

The hepatic schistosomula (10 per well, 5 male and 5 female) were cultured in 24-well plates in medium 841 (RPMI 1640 with 10% fetal bovine serum, 1 μM 5-hydroxytryptamine, 0.5 μM hypoxanthine, 1 μM hydrocortisone, 0.2 IU/mL insulin, 0.1% lactalbumin hydrolysate, 100 IU/mL penicillin, and 100 μg/mL streptomycin) [37] and 1% mouse erythrocytes at 37 °C and 5% CO_2_. Anti-SjCL1 polyclonal antibodies and normal rabbit IgG antibodies were added to the wells. The medium was replaced every two days, with three wells designated for each group. Parasites were collected and measured after 10 days *in vitro*. Imaging was performed using an ECLIPSE E100 (Nikon, Tokyo, Japan). The length of the parasites was measured using ImageJ software (https://imagej.nih.gov/ij/index.html).

### Statistical analysis

The data were analyzed using GraphPad Prism 5.0 (GraphPad, San Diego, CA) and Microsoft Excel 2010. One-way ANOVA with post hoc Tukey’s tests were used to compare between two groups. A *p*-value less than 0.05 was considered statistically significant.

## Results

### Sequence characteristics of schistosome cathepsin L proteases

**Fig 1:**
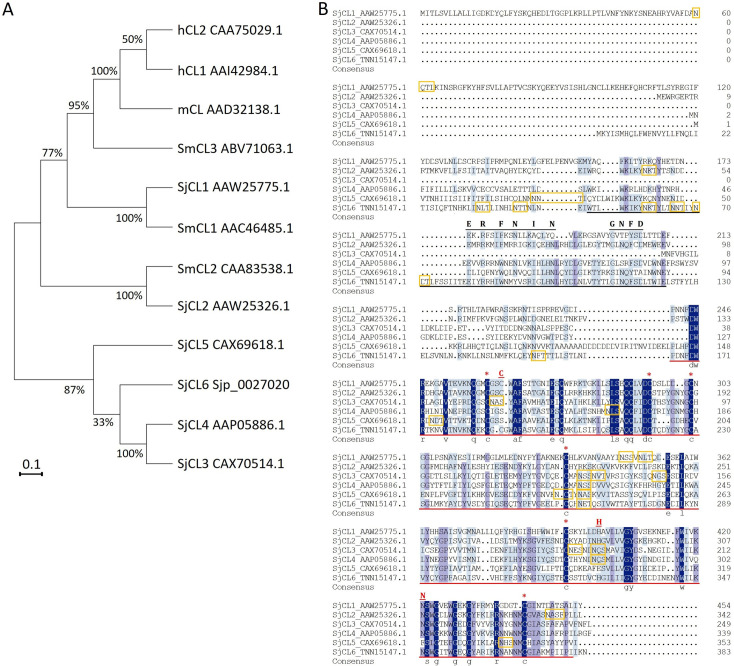
Homology trees and multiple sequence alignment of cathepsin L protease sequences. (A) Phylogenetic tree constructed with amino acid sequences of Cathepsin L proteases from two schistosome species and two host species. The scale of 0.1 is shown below the tree. The frequency for each sequence is shown in bracket before the names. (B) Multiple sequence alignment of SjCLs. Dark Blue, violet, light blue and white indicated 100%, ≥ 75%, ≥ 50% and 0% identity, respectively. Type I-29 protease inhibitor is underlined in black. ERFNIN and GNFD motifs present in the propeptides are overlined with amino acid residues. Peptidase_C1 domain is underlined in red. The catalytic triad residues (C, H and N) are overlined with amino acid residues highlighted in red. Six cysteines forming three putative disulfide bonds that are present the catalytic domain are marked by red asterisk. Putative glycosylation sites are highlighted with yellow boxes. CL: cathepsin L, SjCL: *Schistosoma japonicum* CL, SmCL: *Schistosoma mansoni* CL, hCL: *Homo sapiens* CL, mCL: *Mus musculus* CL.

Our previous work on *S. japonicum* degradome using whole-genome microarray analysis identified 6 schistosome cathepsin L-like proteases showing up-regulated expression in hepatic schistosomula and adult worms [31]. These proteases were termed as *S. japonicum* cathepsin L1 (SjCL1, GeneBank ID: AAW25775.1), SjCL2 (AAW25326.1), SjCL3 (CAX70514.1), SjCL4 (AAP05886.1), SjCL5 (CAX69618.1) and SjCL6 (TNN15147.1), respectively. To date, three cathepsin L proteases have been identified in *S. mansoni*, SmCL1 (AAC46485.1), SmCL2 (CAA83538.1) and SmCL3 (ABV71063.1) [18–21]. The sequence variation of the schistosome CLs and their orthologs from human (hCL1, AAI42984.1, hCL2, CAA75029.1) and mouse (mCL, AAD32138.1) were analyzed. The results showed that the sequences of SjCL1 and SmCL1 are highly homologous, and are more closely related to their host orthologs (Fig 1A). The amino acid composition of schistosome CLs was further analyzed. Among the 6 SjCL proteins, SjCL1 has the longest amino acid sequence (Fig 1B). Similar to SmCL1 and SmCL2, the catalytic triad (Cys, His, Asn) is conserved in SjCL1 (Cys_264_, His_400_, Asn_421_), SjCL2 (Cys_151_, His_281_, Asn_308_) and SjCL6 (Cys_188_, His_326_, Asn_348_), which is essential for their peptidolytic activity. The mature (catalytic) domain has three putative disulfide bonds typical of other cathepsin L enzymes formed by six cysteines [19]. In contrast, SjCL3, SjCL4 and SjCL5 lack these catalytic residues and are likely inactive homologs. Like other cathepsin L proteases, the propeptide of SjCLs (with the exception of SjCL3) contain both a characteristic Ⅰ29 protease inhibitor motif [38] and a variant of the so-called ‘ERFNIN’ motif [39]. These ERFNIN variants include ERFNAQ in SjCL1, ERFRAQ in SjCL2, ERWNIN in SjCL4, DQWNIN in SjCL5, and ERWYIN in SjCL6 (Fig 1B). Furthermore, these SjCLs all possess the GNFD motif (Fig 1B), which is involved in intramolecular processing of Clan CA proteases [40]. Notably, SjCL3 differs fundamentally as it lacks a propeptide entirely and displays the shortest amino acid sequence among the family members. The six SjCL isoforms possess varying numbers of putative glycosylation sites: SjCL1 (3 sites), SjCL2 (2 sites), SjCL3 (6 sites), SjCL4 (4 sites), SjCL5 (5 sites), and SjCL6 (7 sites). The amino acid composition of schistosome CL1, hCL1 and mCL was also analyzed. The Peptidase-C1 domain of SjCL1 has 92.06% identity with SmCL1, yet shares only about 42% identity with hCL1 and mCL (S1 Fig).

### SjCL1 immunization provides partial protection against *S. japonicum* infection in a murine model

**Fig 2:**
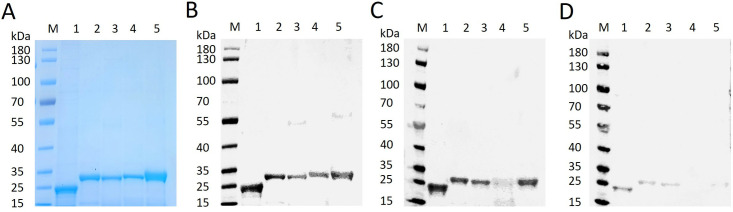
Detection of recombinant protein of SjCLs by SDS-PAGE and western blot. Recombinant proteins of SjCL1-5 were resolved in 12% SDS-PAGE and stained with Coomassie brilliant blue (A), and analyzed by western blot using a mouse monoclonal antibody against the His-tag (B), or a mixture of serum samples (in equal volume) from 6 *S. japonicum*-infected BALB/c mice (C) and 5 *S. japonicum*-infected rabbits (D) collected at 42 dpi, respectively. Lane M: Marker, Lane 1: SjCL1, Lane 2: SjCL2, Lane 3: SjCL3, Lane 4: SjCL4, Lane 5: SjCL5.

**Fig 3:**
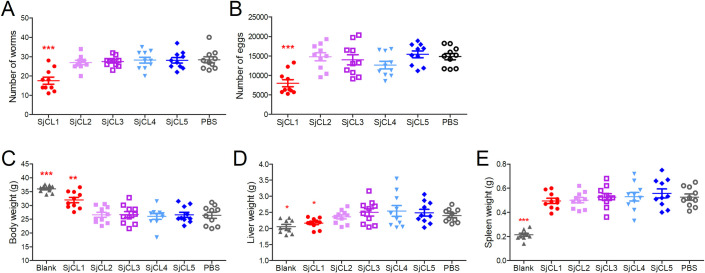
Evaluation of the immunoprotective effects of SjCLs in a murine model. BALB/c mice (n = 10 per group) were immunized with recombinant SjCL1, SjCL2, SjCL3, SjCL4, SjCL5, respectively, or with PBS as control. Following the final immunization, the mice were challenged with cercariae. The blank group (n = 10) consists of mice immunized with PBS but without cercariae challenge. The adult worm burden (A) and liver egg burden (B) were assessed at 42 dpi. for the SjCL1-, SjCL2-, SjCL3-, SjCL4-, SjCL5-immunized groups and PBS control group. The weight of the bodies (C), livers (D) and spleens (E) were measured for all the groups at 42 dpi. Data are presented as mean ± SD. Comparisons were performed between diffident groups with the PBS group, *, *p* < 0.05; **, *p* < 0.01; ***, *p* < 0.0001. The results are representative of two independent experiments.

His-tagged fusion proteins of the mature form of SjCL1–5 were successfully expressed in *E. coli* and isolated from inclusion bodies under denaturing conditions (Fig 2A). The recombinant proteins were further confirmed by western blotting with an anti-His tag mouse monoclonal antibody (Fig 2B). Western blot analysis revealed that rSjCL1, rSjCL2, rSjCL3, and rSjCL5 could be recognized with sera obtained from mice infected with *S. japonicum*, whereas only the former three could be recognized by sera from *S. japonicum* infected New Zealand rabbits (Fig 2C-D). To assess the potential immunoprotective role of SjCLs, BALB/c mice were immunized with rSjCL1, rSjCL2, rSjCL3, rSjCL4 and rSjCL5, respectively, followed by cercariae challenge. Two independent experiments were carried out. The results showed that only the immunization with rSjCL1 conferred partial protection to mice against *S. japonicum* infection, leading to a reduction of 34.9% to 38.0% in worm burden and a decrease of 46.2% to 48.3% in egg burden (Fig 3A-B, S2 Table), while no protective effects were observed for the other four proteins. In addition, rSjCL1 immunization effectively mitigated body weight loss (Fig 3C, S2 Table) and hepatomegaly (Fig 3D, S2 Table) in the challenged mice; however, it had no effect on splenomegaly (Fig 3E, S2 Table).

### SjCL1 was mainly expressed in hepatic schistosomula and adult worms

**Fig 4:**
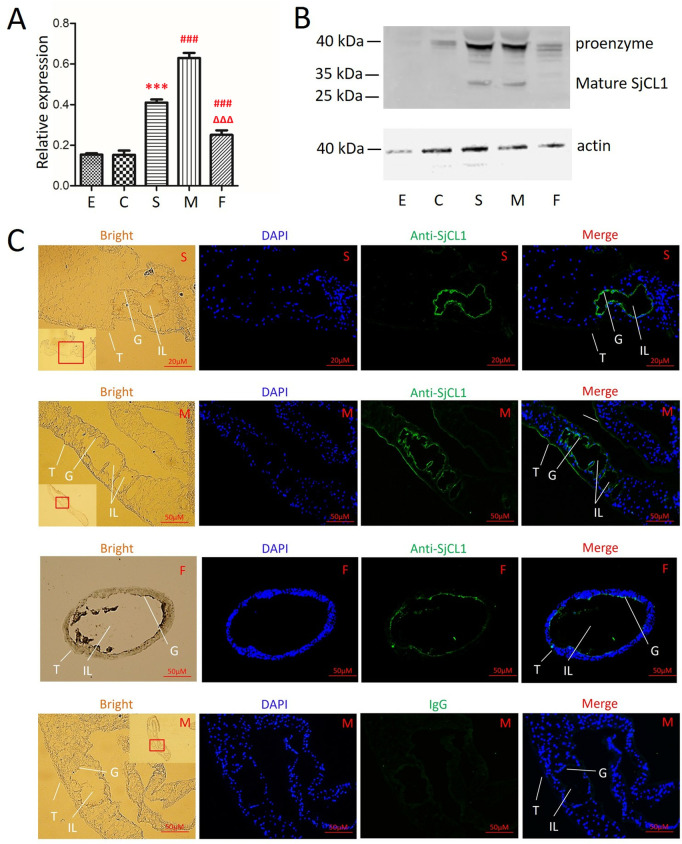
Expression of SjCL1 in parasites at different developmental stages. (A) Relative mRNA expression of *SjCL1* in the five developmental stages of *S. japonicum* was detected by qRT-PCR. Data are presented as mean + standard deviation. ***, ###, and ΔΔΔ indicate the comparisons with *SjCL1* expression levels in eggs, hepatic schistosomula, and adult males, respectively with *p* < 0.001. (B) Expression of SjCL1 and Actin in the five developmental stages of *S. japonicum* were evaluated by western blot. (C) Cryosections of hepatic schistosomula, male adults (longitudinal sections) and female adults (cross sections) were incubated with rabbit anti-SjCL1 polyclonal antibodies, and male adults was incubated with normal rabbit IgG as control, followed by Alexa Fluor 555 donkey anti-rabbit IgG (green fluorescence). The samples were subsequently counterstained with DAPI (in blue). Representative images from three independent experiments are presented, with a minimum of three worms analyzed for each developmental stage in every experiment. E: eggs, C: cercariae, S: hepatic schistosomula, M: adult males, F: adult females. T: tegument, G: gastrodermis, IL: intestinal lumen.

The transcriptional expression of *SjCL1* across different developmental stages/sexes was analyzed by qRT-PCR, revealing the highest expression level in adult males. In addition, the *SjCL1* expression level was higher in hepatic schistosomula and female worms, compared to the other developmental stages (eggs and cercariae) (Fig 4A and S3 Table). To detect the naïve SjCL1 expression and location, polyclonal rabbit antibodies against the rSjCL1 were prepared. This antibody specifically recognizes SjCL1, but not the other four SjCLs (S2 Fig). The protein extracts from different developmental stages of the parasites were analyzed by western blotting, with two bands recognized (Fig 4B). The ~ 40 kDa band appeared to represent the proenzyme form, while the ~ 30 kDa band appeared to indicate the mature SjCL1. The molecular weight of these two native forms of SjCL1 were similar to the predicted sizes of proenzyme (50.0 kDa) and mature enzyme (24.2 kDa). Both forms of SjCL1 were predominantly expressed in hepatic schistosomula and male adults, with low expression observed in cercariae and female adults (Fig 4B). Immunofluorescence assays revealed that SjCL1 is localized along the intestinal intima of hepatic schistosomula, as well as male and female adults, with expression of SjCL1 also observed on the tegument of male adults (Fig 4C).

### SjCL1 was necessary for normal parasite growth

**Fig 5:**
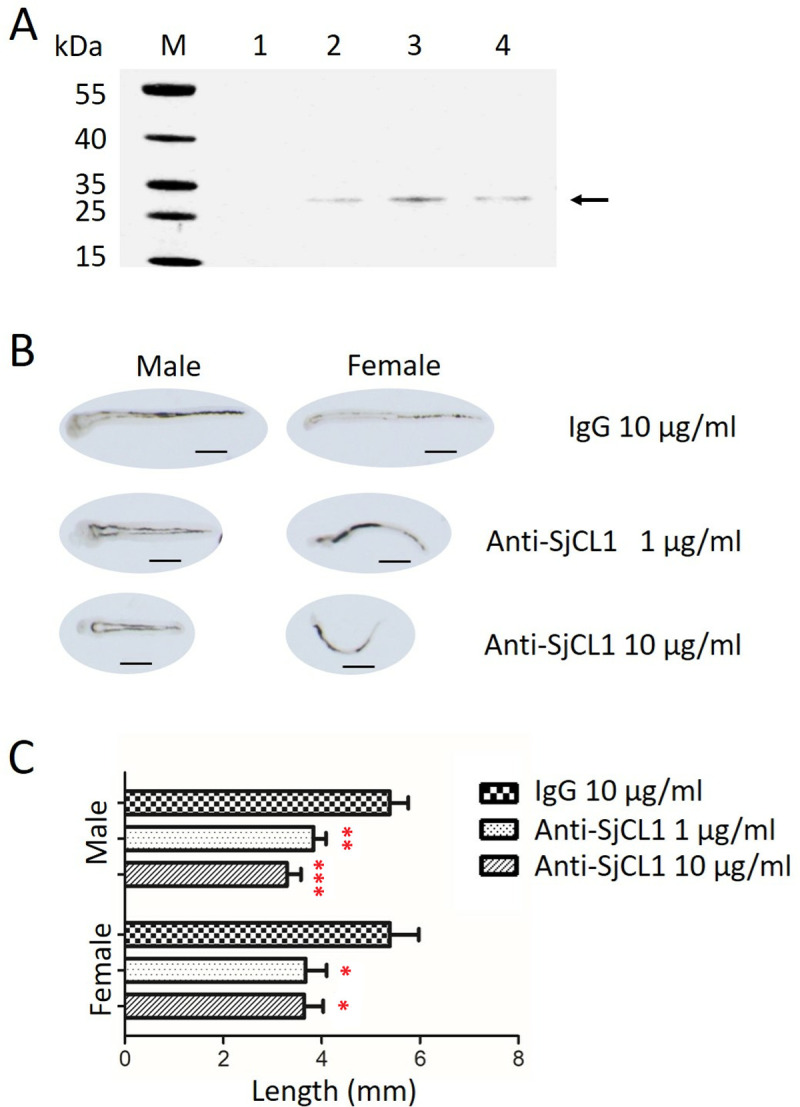
SjCL1 is necessary for normal parasite growth *in vitro.* (A) Schistosome parasites obtained from infected mice were cultured *in vitro* for 8 h. The supernatant was collected and analyzed by western blot using anti-SjCL1 polyclonal antibodies. M: markers; Lane 1, RPMI 1640; lane 2, hepatic schistosomula; lane 3, adult males; lane 4, adult females. (B) Representative hepatic schistosomula cultured *in vitro* with anti-SjCL1 polyclonal antibodies or a rabbit IgG control for 10 days. Scale bar indicates 1.0 mm. (C) Length analysis for the worms cultured under the diffident conditions. Data are presented as mean + SD. *, *p* < 0.05; **, *p* < 0.01; ***, *p* < 0.0001. The results are representative of two independent experiments.

Cysteine peptidases have been identified as the primary constituents of ES products of cercariae, lung-stage schistosomula, and adult worms of *S. mansoni* [41–43], *S. japonicum* [44,45] and *S. haematobium* [46]. Western blot analysis revealed that the mature form (~30 kDa), but not the proenzyme form (~40 kDa) of SjCL, was detectable in the ES products of hepatic schistosomula, and both male and female adults of *S. japonicum* (Fig 5A), indicating that SjCL1 has been processed into its active form prior to being secreted into the gut. To investigate the potential role of SjCL1 in schistosomula growth, an *in vitro* antibody-mediated inhibition assay was performed. Hepatic schistosomula were cultured with specific antibodies targeting SjCL1. After 10 days of incubation, the parasites cultured with anti-SjCL1 antibodies at a concentration of 1 μg/mL exhibited a significant reduction in length compared to those cultured with control IgG, indicating the efficacy of anti-SjCL1 antibodies in inhibiting parasite growth. However, increasing the concentration of anti-SjCL1 antibodies to 10 µg/mL did not enhance this inhibitory effect (Fig 5B-C and S4 Table). Nevertheless, the worms treated with anti-SjCL1 antibodies were viable and developed intestinal heme pigmentation, which is indicative of hemoglobin digestion. These results indicate that SjCL1 is essential for the normal growth of the parasite, though may not be required for hemoglobin digestion.

## Discussion

To achieve sustainable and integrated control of schistosomiasis, comprehensive measures are required and efficacious vaccines could play a key role in this scope. In Science “Unfilled vials” feature, schistosomiasis vaccine was ranked 7th among the top 10 ‘shots’ that require urgent development, based on R&D priority considering feasibility and need [47]. Although a number of candidate schistosome antigens have been characterized and evaluated over the past decades, only four have currently entered human clinical trials. These include *S. haematobium* 28-kD glutathione S-transferase (rSh28GST) [8], *S. mansoni* 14-kDa fatty acid-binding protein (Sm14) [9], *S. mansoni* tetraspanin-2 (Sm-TSP-2), [10] and the large subunit of *S. mansoni* calpain (Sm-p80) [11]. Notably, no vaccine for schistosomiasis japonica has reached the clinical research phase.

Adult schistosome worms can reside in the mesenteric or pelvic veins of the mammalian host for decades and are highly refractory to blood-borne immune defence elements. The early stage schistosome larvae are regarded as vulnerable targets for disrupting the development and reproduction of the parasites, which can result in reduced egg production, a fact closely associated with schistosomiasis-related pathology [48]. In addition, it has been demonstrated that the attrition of schistosomes primarily occurs in the early stage following immunization with radiation-attenuated vaccines [49,50]. Therefore, migrating schistosomulum is considered as the primary target for vaccine development [51], with the key proteins associated with this developmental stage representing a promising array of anti-schistosomal vaccine candidates.

Cathepsin L and cathepsin B-like proteases are the principal cysteine proteases identified in *S. mansoni* and *S. japonicum*, and play crucial roles in the life cycle of the parasites. For example, SmCB1 is a gut-associated protease critical for the digestion of host blood proteins, a key process for nutrient uptake. The protein has been recognized as a potential drug target [15]. RNA interference of SmCB1 in larval schistosomes reduced SmCB1 enzyme activity and led to growth retardation [52]. SjCB2, predominantly found in the acetabular glands and their ducts of cercariae, has been indicated to facilitate skin invasion by degrading the major proteins of the epidermis and dermis [26]. The native SmCL1, located on the tegument of male schistosomes and within the digestive tracts of adult worms, is capable of degrading host haemoglobin [19]. In addition, SmCL3, a protease localized in the adult gastrodermis, can hydrolyze host serum albumin and haemoglobin [21]. Previously, we identified 21 cathepsin genes in the *S. japonicum* degradome, including six schistosome cathepsin L-like proteases [31]. In this study, we characterized the *S. japonicum* cathepsin L protease, SjCL1. Sequence analysis revealed SjCL1 shares high homology with SmCL1, featuring conserved catalytic residues within the Peptidase_C1 domain, along with characteristic ‘ERFNIN’ variant and GNFD motif in the Ⅰ29 protease inhibitor motif. Notably, the Peptidase-C1 domain of SjCL1 exhibits only ~ 42% sequence identity with CLs from mammalian hosts, indicating that vaccines targeting this domain are less likely to interfere with the activity of endogenous cathepsin L in hosts. Immunofluorescence assays demonstrated that SjCL1 is expressed along the intestinal intima of hepatic schistosomula and adult worms, as well as on the tegument of male adults. Functionally active SjCL1 was detected in the ES products from hepatic schistosomula, male and female adults of *S. japonicum.* SjCL1 is essential for the early growth of the parasite, as evidenced by the significant growth inhibition observed in hepatic schistosomula treated with anti-SjCL1 antibodies.

Cathepsins are considered as important targets for novel anti-schistosome chemotherapy and/or immunoprophylaxis due to their roles in nutrient uptake, tissue invasion, and migration. The administration of K11777, a vinyl sulfone cysteine protease inhibitor capable of targeting SmCB1, resulted in a significant reduction in parasite burden during *S. mansoni* infection [53]. In addition, azadipeptide nitriles have been found to be superior inhibitors of SmCB1 over their parent carba analogs, and demonstrated a strong lethal effect on schistosome parasites *in vitro* [54]. By screening a library of peptidomimetic vinyl sulfones against SmCB1, Jilkova et al identified the most potent SmCB1 inhibitors to date and provided structural insights for the rational design of next-generation SmCB1 inhibitors as potential therapeutic agents for schistosomiasis [55]. Notably, treatment with K11777 at early infection stages, e.g., at the time of parasite migration through the skin to lungs (1–14 dpi) resulted in parasitologic cure, evidenced by the cessation of egg production in the majority of experimentally infected mice [53]. However, early treatment of schistosomiasis with chemotherapy is difficult to achieve as patients often seek medical attention only after being infected for a prolonged period.

Vaccines have unique advantages in disease prevention and early intervention. In this study, we demonstrated that immunization with rSjCL1 effectively reduces worm and egg burdens, while preventing body weight loss and hepatomegaly in mice challenged with *S. japonicum*, highlighting its potential as a vaccine candidate against schistosomiasis japonica. However, the protective efficacy of SjCL1, which resulted in a 34.9% ~ 38.0% reduction in worm burden and a 46.2% ~ 48.3% reduction in hepatic egg burden, was notably lower than that of SmCB1. When administered subcutaneously without adjuvants, SmCB1 achieved up to a 66% reduction in worm burden and a 51.3% decrease in liver egg burden in mice challenged with *S*. *mansoni*, the protective efficacy was further enhanced to a 75% reduction in worms and a 58% reduction in liver eggs when SmCB1 was administered with inbuilt adjuvant SG3PDH/PRX-MAP [22]. Vaccination with SmCB1 in combination with *Fasciola hepatica* cathepsin L1 (FhCL1) or SmCL3 and SG3PDH both led to high protection efficacy in worm burden of mice and hamsters challenged with *S*. *mansoni* or *S*. *haematobium*, with concomitant significant reductions in both egg counts and viability [22–25]. Notably, the use of a multiple antigen peptide (MAP) approach, which incorporates SmCB1-derived MAP-1 and cathepsins L-derived MAP-2, induced a highly significant protection (~60% worm burden reduction) in *S. mansoni*-challenged mice [56]. Additionally, immunization with an adenovirus vectored vaccine expressing SmCB1 can also effectively reduce worm burden in *S. mansoni* infected mice, along with an alleviation in liver pathology caused by the infection [57]. Thus, it is essential to conduct more comprehensive research targeting SjCL1, including evaluations of multi-antigen combinations, diverse adjuvant formulations and novel delivery platforms, to determine the most effective immunization strategies against *S. japonicum* infection.

Over the past 2–3 decades, numerous candidate antigens, in addition to cysteine proteases, have been identified and evaluated against schistosome infections. However, there are no vaccines for schistosomiasis japonica available for clinical trials. It is noteworthy that the protective efficacies induced by S*. japonicum* antigens are generally lower than those of *S. mansoni* vaccine candidates [1]. Immunization with Sm14 and Sm-TSP-2 induced a reduction of 67% and 57% respectively, in worm burden in mice challenged with *S. mansoni*, while Sm-p80 vaccination resulted in a 93% worm reduction in baboons, with egg burden reductions of 64% for Sm-TSP-2 and 90% for Sm-p80 [58–60]. However, the highest worm and egg reduction rates generated by *S. japonicum* recombinant antigen SjScP25 were about 50% and 65% in challenged mice [61]. In this study, the worm and egg reduction rates induced by rSjCL1 were only about 36% and 47%, respectively, similar to the previously investigated *S. japonicum* vaccine candidates, such as paramyosin, triosephosphate isomerase, cytosolic fatty acid-binding protein, 23-kDa integral membrane protein, and 16-kDa surface protein [62–65]. Thus, it is crucial to identify and validate additional candidate antigens to facilitate the development of more effective vaccines against *S. japonicum*. In addition, novel immunological strategies and techniques, such as multiantigen-combined immunization and mRNA vaccine, can be adopted to pursue highly effective vaccines for schistosomiasis japonica.

## Conclusions

In this study, we characterized a novel cathepsin L protease from *S. japonicum*, SjCL1, which exhibits the characteristic features of CL proteases. The protease demonstrated an immunoprotective effect against *S. japonicum* infection in a murine model. The findings here provide new insights for the development of anti-schistosomiasis vaccines targeting cathepsins, which are crucial enzymes involved in the early development of schistosome juveniles in mammalian hosts. However, further investigations are required to ascertain the most effective immunization strategies for this protein, such as dosage optimization, adjuvant formulation and delivery vector validation.

## Supporting information

S1 Table Primers for clone construction. (DOCX)

S2 Table Data of Fig 4A. (XLSX)

S3 Table Data of Fig 5C. (XLSX)

S4 Table Results of hepatic schistosomula cultured in vitro with antibodies. (XLSX)

S1 Fig Multiple sequence alignment of cathepsin L1 proteases from various species. Multiple sequence alignment of CL1. Dark Blue, violet, light blue and white indicated 100%, ≥ 75%, ≥ 50% and 0% identity, respectively. Type I-29 protease inhibitor is underlined in black. ERFNIN and GNFD motifs present in the propeptides are overlined with amino acid residues. Peptidase_C1 domain is underlined in red. The catalytic triad residues (C, H and N) are overlined with amino acid residues highlighted in red. Six cysteines forming three putative disulfide bonds that are present the catalytic domain are marked by red asterisk. CL: cathepsin L, SjCL1: *Schistosoma japonicum* CL1, SmCL1: *Schistosoma mansoni* CL1, mCL1: *Mus musculus* CL1, hCL1: *Homo sapiens* CL1.
(TIF)

S2 Fig Detection of the cross-reactivity of anti-SjCL1 antibodies against all recombinant SjCLs by western blot. Recombinant proteins of SjCL1–5 were resolved in 12% SDS-PAGE and analyzed by western blot with anti-SjCL1 antibodies. Lane M: Marker, Lane 1: SjCL1, Lane 2: SjCL2, Lane 3: SjCL3, Lane 4: SjCL4, Lane 5: SjCL5.
(TIF)
