# Correction: *Schistosoma japonicum* cathepsin L1: A potential target for anti-schistosomiasis strategies

**DOI:** 10.1371/journal.pntd.0013796

**Published:** 2025-12-09

**Authors:** The PLOS Neglected Tropical Diseases Staff

There are errors in the author affiliations. The correct affiliations are as follows:

Xianyu Piao^1^, Yuanlong Wang^1^, Ning Jiang^2,3^, Pengfei Cai^4,5^, Jiamei Duan^1^, Shuai Liu^1,6^, Qijun Chen^1,2,3^, Nan Hou^1,6^

1 NHC Key Laboratory of Systems Biology of Pathogens, National Institute of Pathogen Biology, Chinese Academy of Medical Sciences & Peking Union Medical College, Beijing, China, 2 Key Laboratory of Livestock Infectious Diseases in Northeast China, Ministry of Education, Key Laboratory of Ruminant Infectious Disease Prevention and Control (East), Ministry of Agriculture and Rural Affairs, College of Animal Science and Veterinary Medicine, Shenyang Agricultural University, Shenyang, China, 3 The Research Unit for Pathogenic Mechanisms of Zoonotic Parasites, Chinese Academy of Medical Sciences, Shenyang, China, 4 Molecular Parasitology Laboratory, QIMR Berghofer Medical Research Institute, Brisbane, Australia, 5 School of Biomedical Sciences, The University of Queensland, Brisbane, Australia, 6 State Key Laboratory of Respiratory Health and Multimorbidity, National Institute of Pathogen Biology, Chinese Academy of Medical Sciences & Peking Union Medical College, Beijing, China.

The publisher apologizes for the error.
