# Supplementary material for: Schistosoma japonicum cathepsin L1: A potential target for anti-schistosomiasis strategies
Source: PLoS Negl Trop Dis. 2025 Jul 7;19(7):e0013241. doi: 10.1371/journal.pntd.0013241 (PMC12266431; doi:10.1371/journal.pntd.0013241)
Supplement: S1 Table — (DOCX) [file pntd.0013241.s001.docx]

**S1 Table.** Primers for clone construction.

| **Gene ID** | **Primers (5’-3’)** | **Product length (bp)** |
| --- | --- | --- |
| SjCL1  SJCL2  SjCL3  SjCL4  SjCL5 | Forward: TTCGACTGGAGAGAGAAGGGT  Reverse: ATACCACAAGTACCATCACCTC  Forward : GATTGGCGTGATCATGGTGC  Reverse : GGCATTCGATGCAACTCCA  Forward : GCTCTCTCGCCACCGGAATC  Reverse : TGGTGCAAATGCAAATGATGC  Forward : CCGAGGGATCAAGGTTCATGT  Reverse : ACCTCGTAATATTGGTCGAAAAGC  Forward: TTACCGTTACATTTCGATTGGCG  Reverse : AGGATAATAGGCATAACTGGCA | 552  615  648  609  632 |
