# Supplementary figures and images for: Schistosoma japonicum cathepsin L1: A potential target for anti-schistosomiasis strategies
Source: PLoS Negl Trop Dis. 2025 Jul 7;19(7):e0013241. doi: 10.1371/journal.pntd.0013241 (PMC12266431; doi:10.1371/journal.pntd.0013241)

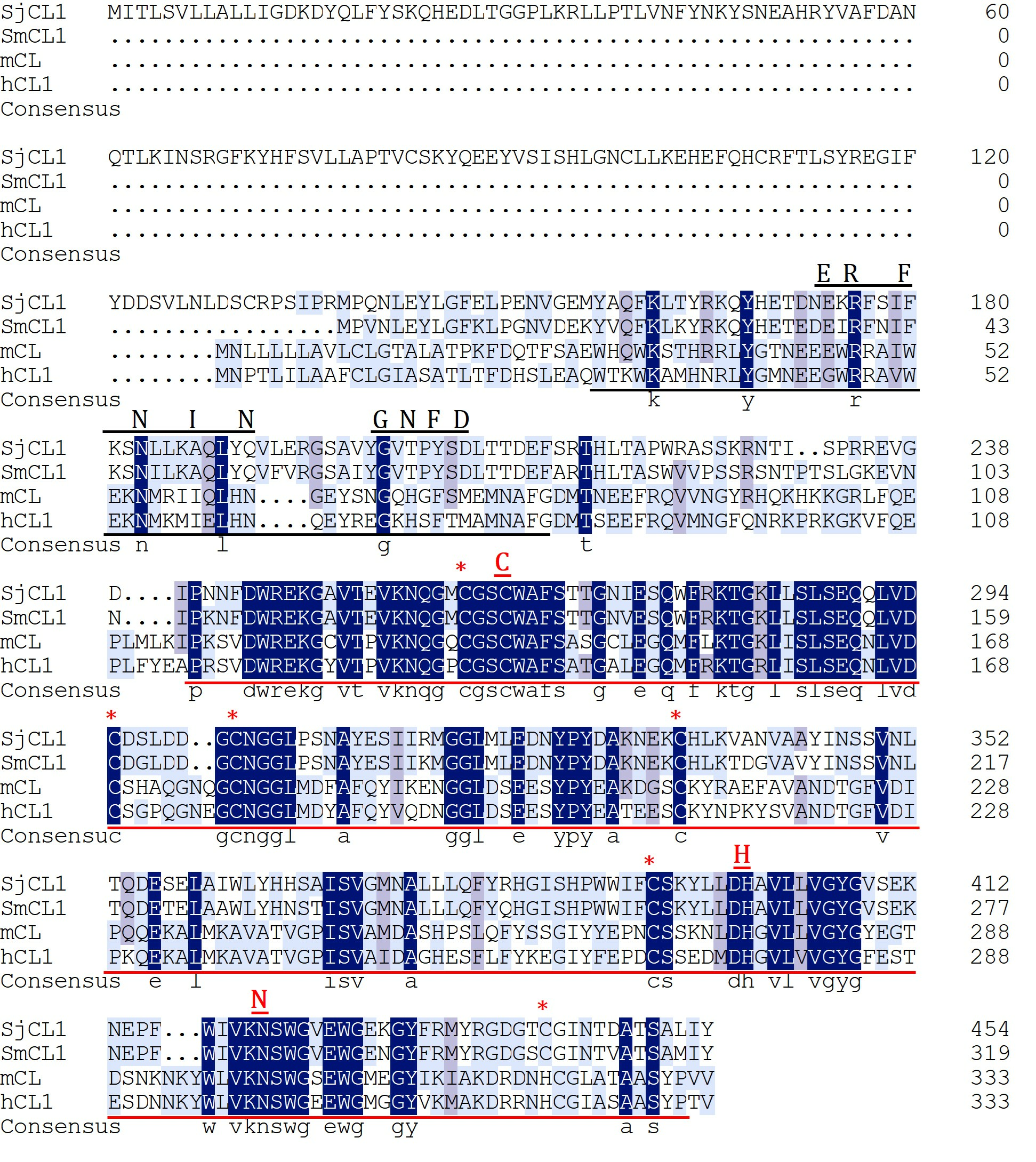

Supplement: S1 Fig — Multiple sequence alignment of CL1. Dark Blue, violet, light blue and white indicated 100%, ≥ 75%, ≥ 50% and 0% identity, respectively. Type I-29 protease inhibitor is underlined in black. ERFNIN and GNFD motifs present in the propeptides are overlined with amino acid residues. Peptidase_C1 domain is underlined in red. The catalytic triad residues (C, H and N) are overlined with amino acid residues highlighted in red. Six cysteines forming three putative disulfide bonds that are present the catalytic domain are marked by red asterisk. CL: cathepsin L, SjCL1: Schistosoma japonicum CL1, SmCL1: Schistosoma mansoni CL1, mCL1: Mus musculus CL1, hCL1: Homo sapiens CL1. (TIF) [file pntd.0013241.s005.tif]

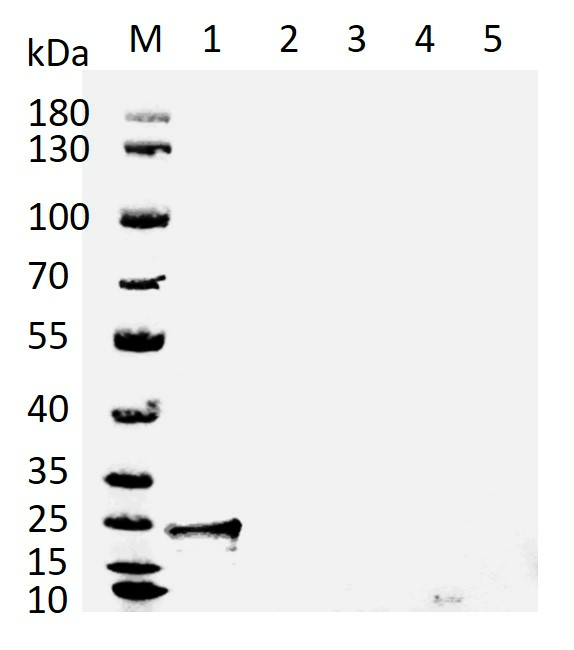

Supplement: S2 Fig — Recombinant proteins of SjCL1–5 were resolved in 12% SDS-PAGE and analyzed by western blot with anti-SjCL1 antibodies. Lane M: Marker, Lane 1: SjCL1, Lane 2: SjCL2, Lane 3: SjCL3, Lane 4: SjCL4, Lane 5: SjCL5. (TIF) [file pntd.0013241.s006.tif]
